# Older adult solitary drinking: associations with subjective and objective cognitive functioning

**DOI:** 10.3389/fnagi.2025.1678121

**Published:** 2026-01-21

**Authors:** Carillon J. Skrzynski, Angela D. Bryan

**Affiliations:** Department of Psychology and Neuroscience, University of Colorado Boulder, Boulder, CO, United States

**Keywords:** drinking alone, objective cognition, older adults, solitary drinking, subjective cognition

## Abstract

**Background:**

Solitary drinking is a pattern of hazardous alcohol consumption that is problematic at any age but is more prevalent in older adults, yet most research focuses on younger samples. Research on solitary drinking and cognition is critical as older adults are more vulnerable to cognitive decline, and cognitive decline is increased by hazardous drinking.

**Methods:**

Using data from a larger project, the present study explored relationships between cognitive function and solitary drinking among 342 individuals 60 + years old (55.56% Female, 89.47% White). Solitary drinking, objective cognition via the Rey Auditory Verbal Learning Test (Rey), and subjective cognition via the Functional Assessment of Cancer Therapy-Cognitive Function (FactCog) questionnaire were assessed at baseline. The FactCog was also completed at a 4-month assessment.

**Results:**

More frequent solitary drinking was correlated with poorer Rey scores and worse scores on the FactCog subscales Perceived Cognitive Abilities (PCA) and Perceived Cognitive Impairment (PCI; ps < 0.05) among older adults who drank alcohol. Older adults who drank alcohol only in social situations had significantly higher baseline Rey learning scores compared to those who did not drink (*p* = 0.04) and higher delayed Recall scores compared to those who drink while alone (*p* = 0.03). They also had significantly higher baseline PCI scores compared to the combined pool of solitary and non-drinking individuals (*p* = 0.046). Finally, PCI averaged across baseline and 4 months was better among the social-only versus solitary drinking group (*p* = 0.03).

**Conclusion:**

Our results expand knowledge of solitary drinking in older adulthood by connecting it to poorer objective and subjective cognitive function.

## Introduction

Solitary drinking is commonly defined as drinking alcohol without the physical presence of others and has been demonstrated to be associated with a myriad of negative sequelae (Skrzynski and Creswell, 2020, 2021). These include increased quantity and frequency of alcohol consumption (Gonzalez and Skewes, 2013) in addition to greater negative outcomes in academic, legal, emotional, and social domains (e.g., poorer grades, experiencing more problems with authorities, negative affect, and loneliness; Tucker et al., 2006; Christiansen et al., 2002; Keough et al., 2015; Skrzynski et al., 2018). Additionally, solitary drinking is predictive of hazardous drinking outcomes including increased alcohol consumption, alcohol use disorder symptoms, and negative-alcohol related consequences (Bilevicius et al., 2018; Creswell et al., 2014; Creswell et al., 2022). Finally, solitary drinking has also recently been shown to be associated with unhealthy characteristics including poorer sleep, higher body mass index scores, and consuming more sugar and less fruits and vegetables (Skrzynski et al., 2024).

While the data are clear that solitary drinking is a particularly problematic form of drinking, most research has focused on younger individuals with relatively few studies on older adults. Importantly, solitary drinking is more prevalent as age increases (Skrzynski and Creswell, 2020, 2021), and adults see similar negative associations with solitary drinking as adolescents and young adults (Skrzynski and Creswell, 2021). Given this demographic reality, solitary drinking research pertaining to older adults is necessary.

One crucial avenue for study within this realm that is particularly relevant to older adults is cognitive functioning. Older adults are vulnerable to cognitive decline and neurodegenerative disorders with a high prevalence of Alzheimer’s disease (AD) and related dementias (Qiu et al., 2009). Subjective cognitive decline is also commonly experienced among older adults (Röhr et al., 2020) and may be an early warning sign for AD and related dementias (Studart and Nitrini, 2016). Of note, excessive alcohol consumption, which solitary drinking is associated with, has been found to increase the risk of objective cognitive dysfunction and dementia and is related to structural and functional brain damage which may lead to cognitive deficits and/or AD while low-to-moderate consumption may be protective against AD and related dementias (Kim et al., 2012; Peters et al., 2008; Rehm et al., 2019; Sachdeva et al., 2016). Unfortunately, little research has been done on subjective cognitive function and alcohol consumption. Given the burden of AD and related dementias, the fact that different patterns of drinking may differentially influence AD and related dementia risk; and the lack of information on subjective cognition and alcohol use, exploring the nuances of different drinking habits on both objective and subjective cognitive functioning among older adults, including associations with solitary drinking, seems especially prudent. However, these relationships have not yet been explored.

The present study sought to address this gap in the literature by conducting the first investigation of the association between solitary drinking and both subjective and objective cognitive functioning among 342 adults aged 60 and over. The first aim examined how proportion of lifetime alcohol use spent in a solitary drinking pattern was related to objective cognitive task performance and self-reported perceived cognitive function. The second aim explored whether individuals who had ever drank alone in their lifetime (*N* = 220), those who had never drank alone (*N* = 78), and a control comparison of non-drinkers (*N* = 44) had baseline differences in these measures and whether they experienced differential changes in perceived cognitive function over the next 4 months (i.e., a time by group interaction). Experience with or greater frequency of solitary drinking was hypothesized to be associated with worse cognitive outcomes.

## Methods

### Participants and procedure

Data came from a larger, longitudinal study examining the impact of cannabis use versus non-use for sleep, pain, and mood problems among older adults (1R01AG066698; PI: Bryan), pre-registered on Clinicaltrials.gov (NCT05188404). Analyses for the current paper are exploratory and were not pre-registered. All data, analysis code, and research materials are available by request to the corresponding author.

Participants were recruited from the greater Boulder-Denver area using mailed flyers and social media posts, and data collection occurred between August 2021 and July 2025. Participants were screened for eligibility via an online survey or over the phone by trained research staff. Criteria included being 60 years or older, able to provide informed consent, no history or family history of psychosis or vertigo, being postmenopausal (where applicable), no engagement in hazardous substance use defined as >8 on the Alcohol Use Disorder Identification Test (Saunders et al., 1993), and screening negative for illicit drug use outside of cannabis at the baseline session. All participants provided written informed consent and were compensated $250 in cash for their participation. The study and all related materials were approved by the University of Colorado Boulder Institutional Review Board.

### Procedure

Participants completed two in-person appointments over the course of 4 weeks followed by three monthly follow-up online surveys. Only data from the first appointment (referred to as baseline hereafter) and last monthly follow-up survey (4 months after baseline, referred to as the 4-month follow-up hereafter) are included in the current analysis. During the baseline, participants completed several questionnaires including information on drinking habits and perceived cognitive functioning as well as a cognitive task (see Measures below); during the 4-month follow-up survey participants completed the same questionnaires on perceived cognitive functioning but did not complete the objective cognitive task.

### Measures

#### Demographics

Participants completed information on age, sex, and race as well as education and relationship status at baseline.

#### Substance use

Alcohol use (i.e., quantity and frequency) was measured via a version of the 30-day Timeline Followback (TLFB; Sobell and Sobell, 1992) modified for online use (O-TLFB; Martin-Willett et al., 2020) during the baseline appointment. A composite alcohol use variable was calculated by multiplying drinks per drinking day by number of days in the last month that participants consumed alcohol for a combined quantity frequency measure (hereafter referred to as QF) and used as a covariate in analyses. Additionally, the first item of the Alcohol Use Disorder Identification Test (AUDIT; Saunders et al., 1993) querying “How often do you have a drink containing alcohol?” with options from 0 “Never” to 4 “Four or more times per month” was utilized to categorize individuals into a non-drinking group (0 “Never” = non-drinker).

#### Solitary drinking

Solitary drinking was assessed at baseline with the following item: “Please think back over all of the times you have consumed alcohol. Please move the bar shown to indicate the percentage of time that your drinking occurred while alone versus with other people from 0 to 100%.” Scale anchor points indicated that 0% meant absolutely no lifetime drinking was spent while alone while 100% meant all lifetime drinking was spent alone (Skrzynski et al., 2018). Correlational analyses included the continuous version of this measure. All other analyses included categorizing individuals who indicated any lifetime solitary drinking (>0%) in a solitary drinking group while those who had never done any lifetime solitary drinking (0%) were categorized into a social-only drinking group. Individuals coded as non-drinking per the AUDIT were a separate group, resulting in three groups in the comparison analyses (i.e., solitary vs. social-only vs. non-drinking).

#### Depression

Given that negative affect is thought to be a primary contributor to drinking patterns, particularly solitary drinking (Skrzynski and Creswell, 2020, 2021; Skrzynski and Creswell, 2021), depression as measured by the depression subscale within the Depression Anxiety Stress Scales (DASS-21; Lovibond and Lovibond, 1995) was included as a covariate in primary analyses excluding correlations. This subscale consists of 7 items with response options ranging from 0 “Did not apply to me at all” to 3 “Applied to me very much or most of the time.” A composite score was calculated and multiplied by two to match the original DASS questionnaire (Lovibond and Lovibond, 1995).

#### Subjective cognitive functioning

The Functional Assessment of Cancer Therapy-Cognitive Function Version 3 (FactCog-V3; Koch et al., 2023) subscales Perceived Cognitive Impairments (PCI) and Perceived Cognitive Abilities (PCA) were used to measure perceived cognitive functioning. These two subscales were included based on scoring recommendations from the Functional Assessment of Chronic Illness Therapy (FACIT) organization (FACIT Organization, 2023). Higher scores for both subscales indicate *better* cognitive functioning. Though its original use was for assessing cognition in the context of cancer, the FactCog version used in the present study has been validated among non-clinical older adult populations (Costa et al., 2018). Participants completed the FactCog V3 at both the baseline and 4 month-follow up.

#### Objective cognitive functioning

The Rey Auditory Verbal Learning Test (Rey, 1964; hereafter referred to as Rey) is a valid and reliable measure of verbal learning and memory commonly used in the aging literature (Brugnolo et al., 2014; Mitrushina et al., 1991; Tierney et al., 1994) and was used to assess objective cognitive functioning. The test includes recalling 8 lists of 15 different words at different time intervals. The total words recalled across the first five trials (i.e., learning score) and the total words recalled after a 20 min delay (i.e., delayed recall score) were included in analyses. The Rey was completed at baseline only as part of a larger battery of cognitive tests within the parent study, but given the lack of variability of most measures, only the Rey was included in the current study.

### Data analysis

All analyses were conducted in R Studio (R Core Team, 2021) and included all available participant data from the larger study. Descriptive analyses were first conducted examining whether there were any demographic or alcohol variables that differed across participants who endorsed any solitary drinking versus those who only endorsed social drinking versus those who did not drink through analyses of variance (ANOVA) tests and chi-square tests for continuous and categorical dependent variables, respectively. Primary analyses included bivariate correlations of Rey scores, PCA, PCI, and solitary drinking (without inclusion of non-drinkers) as well as ANOVAs comparing baseline Rey scores, PCA, and PCI in solitary versus social-only versus non-drinking groups. Finally, mixed effect models using the lme function from the nlme package in R (Pinheiro, Bates, and R Core Team, 2024) were conducted including time, group, and a time by group interaction as predictors of 4-month PCA and PCI. Mixed effect models allowed for random intercepts and random slopes per person. ANOVA and mixed effect models included QF and DASS depression as covariates as well as sex, education, age, and relationship status. The latter were included given that these may be confounders of the relationship between alcohol use and cognition, and there were differences across groups on sex, education, and relationship status (see Results). In cases where *post-hoc* comparisons were probed, the emmeans function from the emmeans package (Lenth, 2024) was utilized and graphs were created using the ggplot function within the ggplot2 package (Wickham, 2016).

## Results

### Descriptives

Descriptive information per group can be seen in Table 1. The overall sample was on average 70 years old (SD = 6.21), and the majority was White (89.47%), in a relationship (68.12%), unemployed^1^ (61.47%), and had a college or advanced degree (85.29%). The sample was generally evenly split between males and females (55.56% female). Individuals in the solitary drinking group spent an average of 32.80% (SD = 26.21) of drinking time alone.

**Table 1:** Demographics and general alcohol use information across solitary vs. social-only vs. non-drinking groups.

Drinking groups	Non-drinking (*N* = 44)	Solitary drinking (*N* = 220)	Social-only drinking (*N* = 78)	*p*-value
Age, m (sd)	69.66 (5.12)	69.95 (6.44)	70.36 (6.19)	0.20
Sex (female), n (%)	30 (68.18)	112 (50.91)	48 (61.54)	**0.05**
Race (White), n (%)	37 (84.09)	197 (89.55)	72 (92.31)	0.36
Education (college or advanced degree), n (%)	32 (72.73)	188 (85.45)	70 (89.74)	**0.02**
Relationship status (in a relationship), n (%)	23 (52.27)	145 (65.91)	65 (83.33)	**<0.001**
Employment status (employed), n (%)	13 (29.55)	86 (39.09)	32 (41.03)	0.51
Drinks per drinking day	0.10 (0.69)	1.34 (0.86)	1.06 (0.90)	**<0.001**
Total drinking days	0.23 (1.51)	11.83 (9.78)	5.53 (7.11)	**<0.001**
QF	1.04 (6.93)	19.11 (21.37)	8.64 (15.61)	**<0.001**
Dass Depression, m (sd)	8.86 (9.62)	6.64 (6.39)	4.28 (5.81)	**<0.001**

QF, Composite quantity frequency measure. Bold values indicate significant *p*-values.

Across the drinking groups, there were differences of sex, relationship status, education status, drinks per drinking day, total drinking days, QF, and depression scores. Based on *post-hoc* pairwise comparisons, there were more females among the non-drinking group compared to the solitary drinking group (*p* = 0.05); there were more individuals in a relationship among the social-only drinking group compared to both solitary drinking (*p* = 0.01) and non-drinking groups (*p* < 0.001); there were more individuals who had a college or advanced degree in the solitary drinking and social-only drinking groups compared to the non-drinking group (ps ≤ 0.05), and as would be expected, there were fewer drinking days and drinks per drinking day in the non-drinking group compared to both social-only and solitary drinking groups (ps < 0.001). Further, those in the solitary drinking group consumed more alcohol, both in terms of quantity and frequency (and thus had higher combined QF values) than those in the social-only drinking group (ps < 0.04). In terms of depression scores, the solitary and non-drinking groups did not differ from each other (*p* = 0.12) while both groups had higher depression scores than the social-only group (ps < 0.03).

### Bivariate correlations

Correlational analyses are shown in Table 2 and did not include individuals in the non-drinking group. More frequent solitary drinking was associated with worse Rey learning scores and PCA at baseline and worse PCA and PCI at follow-up. Notably, Rey learning and delayed recall scores were positively correlated with each other as well as with PCA and PCI at both baseline and 4-month follow-up (i.e., better performance on the *objective* memory task scores were associated with better *perceived* cognitive functioning). PCA and PCI scores were also positively correlated with each other at both timepoints.

**Table 2:** Bivariate correlations across solitary drinking, PCI, PCA and Rey scores.

Variables	Baseline solitary	Baseline PCI	Baseline PCA	Baseline Rey learn	Baseline Rey delay	4 Month PCI	4 Month PCA
Baseline Solitary	-						
Baseline PCI	−0.08	-					
Baseline PCA	−0.12*	0.59***	-				
Baseline Rey Learn	−0.11*	0.17**	0.19***	-			
Baseline Rey Delay	−0.09	0.20***	0.17**	0.79***	-		
4 Month PCI	−0.12*	0.80***	0.51***	0.26***	0.26***	-	
4 Month PCA	−0.14*	0.66***	0.64***	0.25***	0.20***	0.78***	-

Solitary, solitary drinking percentage; PCI, perceived cognitive impairments, PCA, perceived cognitive abilities; Rey Learn, Rey auditory verbal learning test learning score; Rey Delay, Rey delayed recall score. *p<0.05, **p<0.01, ***p<0.001.

### ANOVA comparisons

#### Objective cognitive function

**Figure 1:**
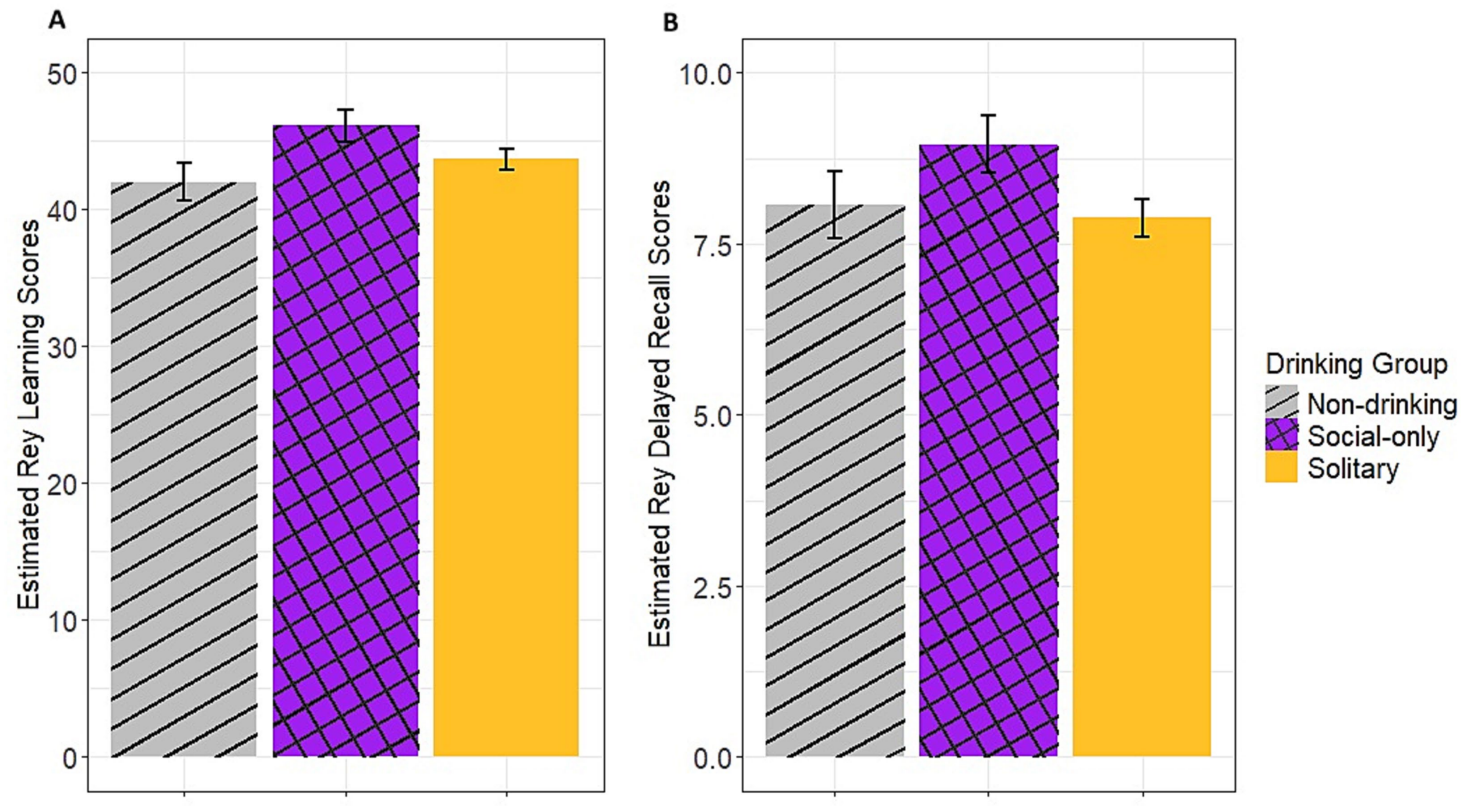
Estimated Rey learning and delayed recall scores across groups. **(A)** Estimated Rey learning scores, **(B)** Estimated Rey delayed recall scores.

Results from the ANOVAs showed a significant main effect of sex [*F*(1,328) = 42.90, *p* < 0.001], age [F(1,328) = 38.48, *p* < 0.001], and group [*F*(2,328) = 5.17, *p* = 0.01] on baseline Rey learning scores. *Post-hoc* pairwise analyses showed a significant pairwise difference whereby the non-drinking group had a lower score [estimated Mean (eM) = 42.00, standard error (SE) = 1.39] compared to the social-only group (eM = 46.10, SE = 1.20, *p* = 0.04) but neither of these groups differed from the solitary drinking group (eM = 43.70, SE = 0.78, ps > 0.11; see Figure 1, panel A). Additionally, females scored higher (eM = 46.60, SE = 0.83) than males (eM = 41.30, SE = 0.99) and older age was associated with lower scores. There were similarly main effects of sex [*F*(1, 329) = 36.04, *p* < 0.001], age [*F*(1,329) = 27.68, *p* < 0.001], and group [*F*(1,329) = 5.01, *p* = 0.01] for the Rey delayed recall scores. The main effects of age and sex followed the same pattern as for the learning scores, but *post-hoc* tests of the group effect indicated that the solitary drinking group had lower scores (eM = 7.88, SE = 0.28) than the social-only drinking group (eM = 8.95, SE = 0.42, *p* = 0.03) while there was no difference between these groups and the non-drinking group (eM = 8.97, SE = 0.49, ps > 0.31; see Figure 1, panel B).

#### Subjective cognitive function

**Figure 2:**
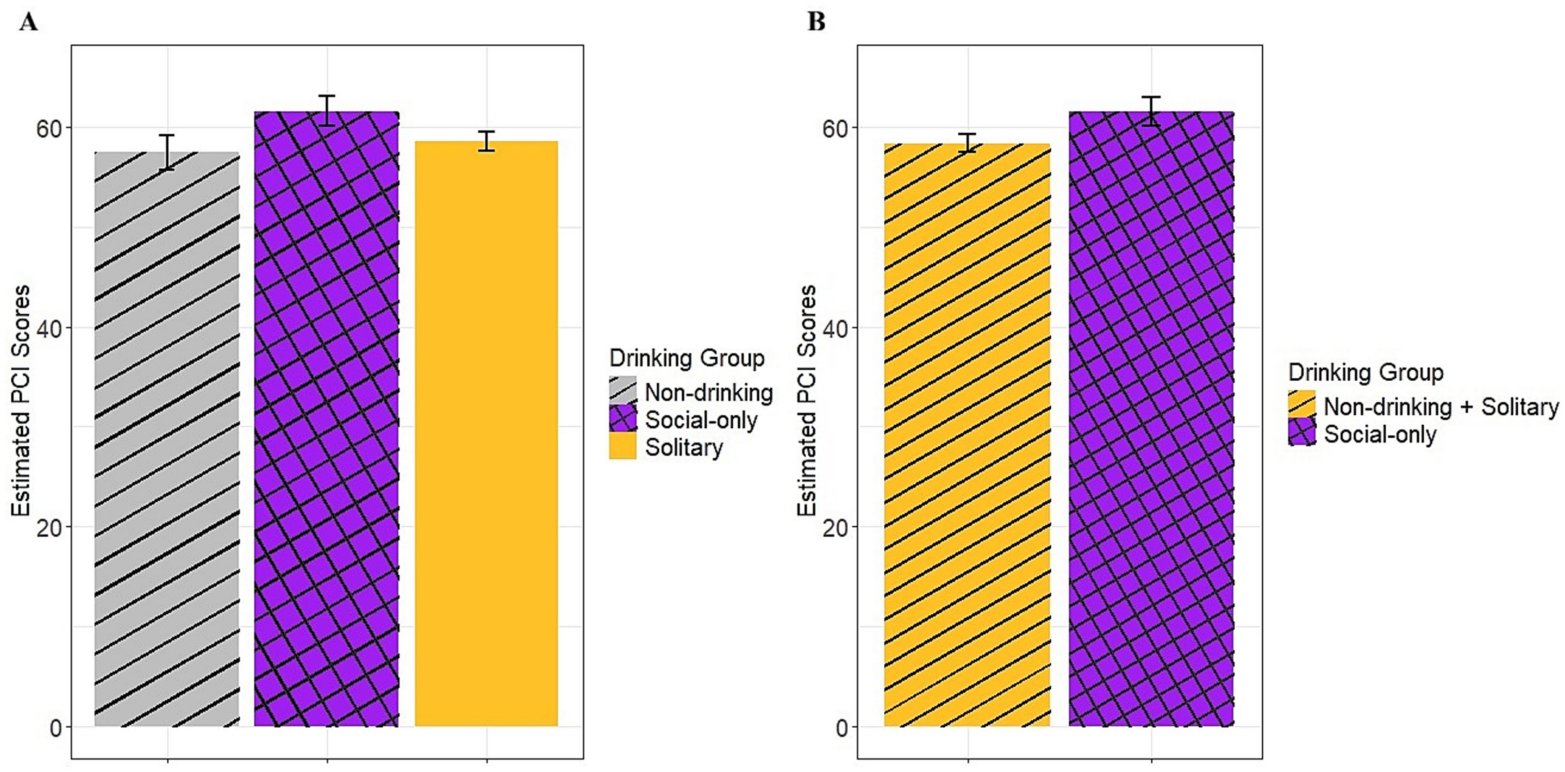
Estimated baseline PCI scores across groups. **(A)** compares all three groups and **(B)**compares the social-only group to the combined solitary drinking and non-drinking groups.

Findings based on PCI scores likewise showed a main effect of group [*F*(2,329) = 4.51, *p* = 0.01; see Figure 2, panel A] but no significant differences emerged based on pairwise *post-hoc* testing. However, the combined solitary and non-drinking groups had significantly lower PCI scores (eM = 58.30, SE = 0.89) compared to the social-only group (eM = 61.50, SE = 1.48; *p* = 0.004; see Figure 2, panel B). Additionally, there was a main effect of depression [F(1,329) = 30.33, *p* < 0.001] with greater depression scores associated with lower PCI scores.

In contrast, the difference on PCA scores did not result in a significant main effect of group (*p* = 0.11). However, like with PCI, there was a main effect of depression [F(1,329) = 19.04, *p* < 0.001]. This association followed the same pattern as with PCI where greater depression scores were associated with lower PCA scores.

### Change in subjective cognitive function over time

**Figure 3:**
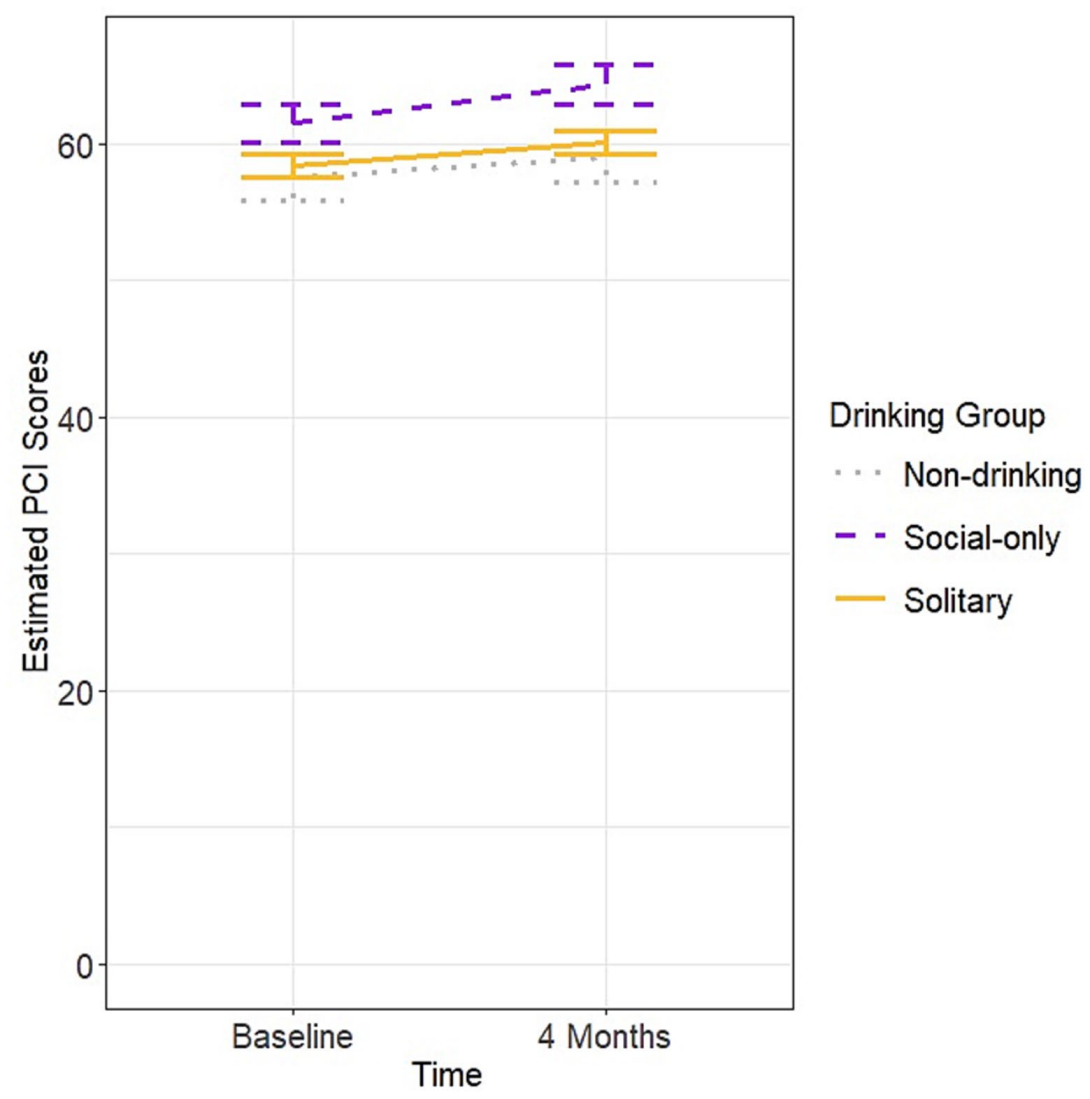
Estimated PCI scores over time by group.

Mixed effect model findings did not result in a significant time by group interaction [F(2,285 = 0.73, *p* = 0.48] on PCI scores but did demonstrate a main effect of group [*F*(2,328) = 4.47, *p* = 0.01]. *Post-hoc* pairwise comparisons indicated that while the non-drinking groups’ scores (eM = 58.30, SE = 1.67) did not differ from those of the solitary drinking group (eM = 59.20, SE = 0.80; *p* = 0.87) or social-only group (eM = 62.90, SE = 1.35; *p* = 0.06), the solitary drinking group had worse PCI scores than the social-only group (*p* = 0.03; see Figure 3). Additionally, there was a main effect of depression [*F*(1,328) = 34.02, *p* < 0.001] which again demonstrated that higher depression scores were associated with worse PCI scores. There was also a main effect of age [F(1,328) = 4.63, *p* = 0.03] with older age associated with worse PCI scores. The model examining PCA did not find a significant interaction between time and group [F(2,285) = 1.94, *p* = 0.15] nor a main effect of group [F(2,329 = 2.39, *p* = 0.09] but again found a main effect of depression [F(1,329 = 34.84, *p* < 0.001] with the same pattern as in the PCI models.

## Discussion

The present study is the first to examine relationships between solitary drinking and cognitive functioning, both objective and subjective, among older adults. Findings generally supported hypotheses. Solitary drinking was correlated with worse scores on objective and subjective cognitive measures (i.e., baseline Rey learning and delayed recall scores and PCA scores, 4 month PCA and PCI scores), and compared to those who only engaged in social drinking, individuals who drank alone had worse baseline Rey delayed recall scores and those who drank alone and who did not drink had worse baseline PCI scores. Additionally, while there was no interaction in the mixed effect models, in the case of the PCI results, the main effect of group revealed a difference between the social-only drinking group and the solitary drinking group, with worse scores among the solitary drinking group.

In terms of the group differences, it is interesting that those in the solitary drinking group did not differ from the non-drinking group in any analyses, and in the case of baseline PCI cognitive functioning, it was only when solitary drinking and non-drinking groups were combined that there were differences from the social-only drinking group. It is also noteworthy that Rey learning scores were worse between the social-only and non-drinking groups and for PCI scores averaged across baseline and 4 months as well as the baseline Rey delayed recall, scores were worse for those in the solitary drinking group relative to the social-only drinking group. These findings may indicate a potential inverted u-shaped curve such that solitary drinking and non-drinking may both be associated with poorer cognitive function relative to social-only drinking, and this may suggest that both signal risk in this domain. Interestingly, similar patterns have been found in studies on general alcohol consumption in prior literature with no alcohol consumption and higher quantities of consumption associated with lower cognitive abilities compared to low-to-moderate amounts of alcohol consumption (Cansino et al., 2025; Kim et al., 2012; Müller et al., 2013; Peters et al., 2008; Piumatti et al., 2018; Rehm et al., 2019; Sachdeva et al., 2016; Wardzala et al., 2018). As the solitary drinking group were heavier drinkers than the social-only drinking group and the non-drinking group inherently consumed no alcohol, our findings seem to generally align with these prior studies.

Also interesting is that there was no interaction between time and group in the PCI mixed effect model. One potential reason for the lack of a significant interaction may be that 4 months is not long enough to see meaningful change in cognitive functioning, In fact, there was no main effect of time in either mixed effect model indicating no changes from baseline to 4 months later. Another potential explanation is that the sample had high cognitive functioning to begin with, potentially also limiting our ability to demonstrate changes. Indeed, the mean score on the Rey learning trials for the whole sample (M = 45.70, SD = 9.65) was higher than the average score of individuals within the 70–79 age bracket (M = 37.10, SD = 7.5) based on meta-data from several studies (Schmidt, 1996). Additionally, according to FactCog normative data (Lange et al., 2016), our average sample scores put participants somewhere between the 50 and 60th percentile for PCI and 60 and 70th percentile for PCA scores, respectively. Interestingly, the difference in PCI scores found across the solitary/non-drinking group score and the social-only drinking group score aligns with different percentiles, suggesting clinically meaningful discrepancies in subjective functioning across these groups (i.e., solitary/non-drinking groups were between the 40th and 50th percentile for PCI while the social-only drinking group score was around the 70^th^ percentile). The current study is not without its limitations, and study findings should be considered in light of these. One limitation includes that the sample was a convenience sample and mostly white and well-educated. This may limit generalizability, and research including more racially and social-economically diverse samples is indicated. Another limitation is that the sample was generally quite healthy in terms of behavioral health metrics. Specifically, our eligibility criteria precluded hazardous alcohol use and most (80%) of the sample with physical activity data indicated that they engaged in moderate intensity exercise 3 or more days a week. Given that empirical work indicates a positive association between physical activity and cognitive functioning (Gomes-Osman et al., 2018) and a negative association between hazardous alcohol use and cognitive functioning (Kim et al., 2012), this could have influenced study findings. Although we included alcohol consumption in our analyses as a covariate to address the former point, we were unable to include physical activity status given the amount of missing data (i.e., 53 individuals did not provide this information) causing concern this would affect model findings. Other limitations that should be noted are that the objective measure for cognition was not assessed at the follow-up timepoint, there were only 4 months between timepoint assessments, and we utilized a measure of lifetime (versus, for example, past month) solitary drinking. Future work may wish to include longitudinal objective measures of cognitive functioning as well as more proximal measures of solitary drinking in addition to assessing cognition over a longer period of time. Finally, we cannot definitively comment on a causal relationship between solitary drinking and cognition given that we examined objective cognitive performance cross-sectionally, and we did not find a time by group interaction on subjective cognition in our models spanning baseline to 4 months. It may be that declines in objective and subjective cognition lead to individuals engaging in more solitary drinking. Further, our objective cognition measure is specific to verbal learning and memory so it is unclear if this would extend to other cognitive domains.

These limitations are tempered by a few notable strengths of the study. In particular, we included a large sample size of over 300 individuals providing adequate power for analyses, and some variability across variables of interest. Additionally, the inclusion of depression, quantity and frequency of drinking, age, education, and relationship status as covariates was a strength. Indeed, the inclusion of an alcohol use quantity and frequency covariate specifically helps to delineate how social vs. solitary context of drinking, rather than alcohol use generally, may be related to cognitive function. Similarly, though relationship status and depression are not direct measures of social disconnectedness/discontent (e.g., loneliness, living alone), both are negatively related to cognition (Liu et al., 2019; Wang and Blazer, 2015) such that they may serve as proxies and partially control for confounding in this area. However, actual measures of loneliness and being physically alone while drinking may be more informative and future work including these measures in studies of solitary drinking and cognition would be useful.

In sum, the present study found that solitary drinking was related to poorer objective and subjective cognitive functioning via bivariate correlations. Additionally, older adults who engaged in either solitary drinking or did not drink at all demonstrated lower baseline subjective cognitive performance compared to older adults who engaged in social-only drinking. Similarly, those who engaged in solitary drinking had lower averaged subjective cognitive scores and baseline delayed recall than those in the social-only drinking group. Overall, this study provides novel, though preliminary, insight into the phenomenon of solitary alcohol consumption and cognition among older adults, suggesting that solitary drinking may be a problematic form of drinking for older adults within the cognitive domain. However, future research is needed to continue to shed light on this harmful drinking behavior. In particular, more work including samples with varied patterns of physical activity, alcohol consumption, and cognitive functioning utilizing diverse subjective and objective measures over longer periods of time are indicated.

## Data Availability

The raw data supporting the conclusions of this article will be made available by the authors, without undue reservation.

## Footnotes

1 Employment status was queried by asking “Are you employed?” with the following response options: “Full- time employed (work 30 or more hours per week)”; “Part-time (work less than 30 h per week)”; “Unemployed, disabled, retired, other”; “Full-time student”; “Homemaker/stay at home mom or dad.” To ensure adequate expected cell counts for chi-square analyses, this variable was recoded into employed (full or part-time employment) vs. unemployed (any other answer).
